# Nitric oxide and inducible nitric oxide synthase levels in EE and NERD patients 

**Published:** 2022

**Authors:** Fatemeh Nejat PishKenari, Durdi Qujeq, Seyed Saeid Mohammady Bonahi, Mehrdad Kashifard, Karimollah Hajian -Tilaki

**Affiliations:** 1 *Student Research Committee, Babol University of Medical Sciences, Babol, Iran*; 2 * Cellular and Molecular Biology Research Center (CMBRC), Health Research Institute, Babol University of Medical Sciences, Babol, Iran*; 3 * Department of Clinical Biochemistry, Babol University of Medical Sciences, Babol, Iran*; 4 * The Internal Clinic, Babol, Iran*; 5 * Department of Internal Medicine, Gastroenterology Division, Ayatollah Rouhani Hospital, Babol University of Medical Sciences, Babol, Iran*; 6 * Department of Biostatistics and Epidemiology, Babol University of Medical Sciences, Babol, Iran*

**Keywords:** Gastro-esophageal reflux disease, iNOS, Nitrite, Nitrate, Non-erosive reflux disease, Erosive esophagitis

## Abstract

**Aim:**

This article aimed to evaluate nitric oxide (NO) and nitric oxide synthase (iNOS) markers in patients with erosive esophagitis (EE) and those with non-erosive reflux disease (NERD) and compare them with the control group.

**Background:**

Gastro-esophageal reflux disease (GERD) is one of the most common disturbances of the upper digestive tract. Inducible nitric oxide synthase (iNOS) is expressed in esophageal adenocarcinoma. NO, the product of this enzyme, has been implicated in the pathogenesis of this condition. Nevertheless, the data on whether iNOS and NO are expressed in the early stages of GERD is conflicting.

**Methods:**

In this study, tissue samples were obtained from fifty-four patients (27 with erosive esophagitis and 27 with non-erosive reflux disease) and 27 controls. Tissue concentrations of nitrite, nitrate, and iNOS were measured using Enzyme-Linked Immune-sorbent Assay (ELISA). The Bradford method was used to determine the protein concentration of samples. The results were analyzed by SPSS software (version 22.0). In multiple comparisons, the Tukey test was performed, and *p* < 0.05 was considered as the level of significance.

**Results:**

Tissue amounts of iNOS were signiﬁcantly higher (*p*= 0.001) in EE patients compared with the control group. There was a significant difference (*p*= 0.01) in this factor between EE patients and patients with NERD. Moreover, tissue levels of nitrite and nitrate were signiﬁcantly higher (*p* = 0.001) in patient groups compared with the control group.

**Conclusion:**

It was observed that NO and iNOS protein were increased in human esophagitis tissue. The results indicated that nitric oxide and iNOS levels are useful and effective markers in the pathogenesis of GERD. While the results are not certain, it is thought that a link exists between the expressions of iNOS and disease progression.

## Introduction

 Gastro-esophageal reflux disease (GERD) is a multi-factorial disorders of the upper digestive tract (1-3) in which the acidic contents of the stomach return to the esophagus (4, 5). In the absence of effective sphincter, increased abdominal pressure causes the return of the acidic contents, and damage to the esophagus, and subsequently GERD disease (6, 7). The mechanisms that encourage the pathogenesis of GERD include mechanical deficiencies of the lower esophageal sphincter, ineffective esophageal clearance, the presence of hiatal hernia, delayed gastric emptying, and increased acid secretion (8). Erosive esophagitis (EE) and non-erosive reflux disease (NERD) are the prevalent phenotypes of GERD (9). Erosive esophagitis is a severe form of GERD and is defined as the existence of a distinguished lesion in the esophagus of patients with or without signs of GERD that have been damaged by the abnormal reflux of gastric acid (10). The majority of GERD patients present with NERD, which is characterized by the absence of macroscopic inflammation in the esophageal mucosa (11, 12). Patients with abnormal acid exposure, with or without symptom-reflux association, in the absence of visible esophageal mucosal injury during upper endoscopy, are considered as cases of true NERD (13, 14). Esophagitis improves in most cases through the re-production of squamous cells at the end of the esophagus (15). If esophagitis continues, however, it can cause Barrett's esophagus (BE), a condition in which the normal stratified lining of the esophagus is replaced with a metaplastic specialized intestinal-type epithelium with goblet cells (16-19). Contrary to the past, when researchers considered the effects of gastric reflux to be due to the direct effect of acid, today some researchers suggest that the complications of reflux are due to the role of the immune system and inflammation. One of the proteins produced in inflammation is nitric oxide synthase. This enzyme produces nitric oxide during the conversion of L-arginine to L-citrulline (20). NOS has three isoforms: nNOS, eNOS, and iNOS. The pro-inflammatory cytokines cause iNOS expression in monocytes/macrophages, neutrophils, and many other cells (21-23). As an unstable free radical, NO plays an important role in immune responses. The final oxidation products of NO are nitrite and nitrate. NO levels represent NOS activity like iNOS, which produces NO in high levels (24, 25). In humans, NO is a signaling molecule in many physiological and pathological processes (26, 27). It can act as either a pro-inflammatory or an anti-inflammatory factor, depending on its concentration (28). It is believed that NO causes vasodilatation in the cardiovascular system. Furthermore, NO is a strong neurotransmitter in the synapse of neurons and helps regulate apoptosis. It is involved in the pathogenesis of inflammatory disorders of the joint, intestine, and lungs (29-31). In many biological systems, it acts as a messenger molecule and affects itself through the production of circular guanosine monophosphate (cGMP). Soluble guanylyl cyclase is the receptor of NO. When these two pair together, GPT converts to cGMP, and then the protein kinase G is activated. All these are signs for the immigration of a cancerous cell, and it is essential to attack tumor cells and metastasis (32). On the other hand, NO can react with O_2_^-^ and create secondary power intermediates like ONOO^-^ and NO_2_ (33, 34), which intervene with their cytotoxic effects through its effect on lipid and protein metabolism, DNA and RNA damage, and post-translational protein changes (35, 36). Enhanced iNOS expression has been reported in different types of cancer (37-39). The goal of the current study was to measure the levels of nitrite, nitrate, and iNOS in biopsy specimens of the esophagus from patients with EE, NERD, and controls. 

## Methods

**Patients**

Fifty-four patients (28 males; 26 females; 44.05 ± 13.70 median age; age range of 18-80 years) undergoing upper endoscopy due to reﬂux symptoms and/or previous information from patients were entered in the current study. Patients were divided into 2 groups: erosive esophagitis and non-erosive reflux disease. There were 27 patients in each of the EE, NERD, and control groups. The severity and frequency of GERD symptoms were assessed using a standardized questionnaire. Entry criteria comprised female or male, age 18–80, able to write informed consent, patients with typical reflux symptoms experiencing symptoms at least three times a week. Typical symptoms of reflux were determined as heartburn and regurgitation. Patients with other symptoms of reflux were not included in this investigation. Exclusion criteria comprised taking NSAIDs, corticosteroids, anti-allergic drugs, or other immunosuppressive drugs, proton pump inhibitors (PPI), or H2 antagonists (at least two months before sampling), having an upper digestive system disease (like cancers, peptic ulcers, polyps, and Barrett’s esophagus) or a mal-absorptive disease (like celiac disease, Crohn’s disease, vasculitis, or ulcerative colitis). 

**Control subjects**

The volunteers who entered this experiment as the control group were from the general population of Mazandaran (including subjects who wanted to have a checkup). Health was determined as physical and social well-being and the lack of any acute or chronic illnesses and no acute or chronic drug use (40). Exclusion criteria comprised micronutrient supplementation, smoking, or pregnancy.

**Ethical Considerations**

Informed written consent was obtained from all patients before endoscopy. This study was approved by the human subjects ethics board of Babol University of Medical Sciences and was conducted in accordance with the Helsinki Declaration of 1975, as revised in 2013. All protocols involving patients and control subjects were confirmed by the Ethics Committee of Babol University of Medical Sciences with the code number (P/J/30/1384, 96/11/04).

**Sampling and Assay**

During endoscopy, a sample esophagus biopsy was taken from each patient. Each sample was then washed in cold phosphate buffered saline (PBS) and was immediately frozen at -80 °C. After sample collection was completed, all biopsy tissues were weighed, and a determined amount of protease inhibitor cocktail and PBS were added for tissue homogenization with an ultrasonic device. Then, the supernatant was isolated by centrifugation to measure the factors. The supernatant was divided into four portions and stored at −20 °C.

**Biochemical Assays**

The Bradford test (41) was performed for each sample to determine the protein content of the tissue. The Bradford method is based on the shift in absorbance maximum of Coomassie Brilliant Blue dye from 465 to 595 nm following binding to denatured proteins in the sample. With this method, the level of protein can be measured by determining the amount of dye, which is determined by measuring the absorbance of the sample at 595 nm.

Tissue iNOS concentrations were measured using the human iNOS assay kit (product code: E0928Hu) provided by Bioassay Technology Laboratory (China) according to the manufacturer’s guides.

Nitrate concentrations were measured spectrophotometrically (Microplate reader, model: RT 2100C, Hamburg, Germany), using the human nitric oxide assay kit (lot: NO1471), provided by Biocorediagnostik Ulm GmbH (Zell Bio GmbH, Germany). To measure nitrite, nitrate reduction was prevented by deleting nitrate reductase from the assay. 

**Statistical Analysis**

SPSS software (version 22.0) was used to analyze the data. One way analysis of variance was applied to test nitrite, nitrate, and iNOS levels in the three groups under study. In multiple comparisons, the Tukey test was performed, and a *p*-value < 0.05 was considered as significant.

Calculate

$\frac{\mathrm{INOS concentration}(U/L)\times \mathrm{Dilution factor}}{\mathrm{The protein concentration of samples}(\mathrm{mg}/\mathrm{ml})\times \mathrm{Dilution factor}}=U/\mathrm{mg protein}$

Nitrite or nitrate (µmol/l) × 1000 → mmol/l → Calculate in the intended volume → Calculate in one gram of tissue. 

## Results

**Figure 1:**
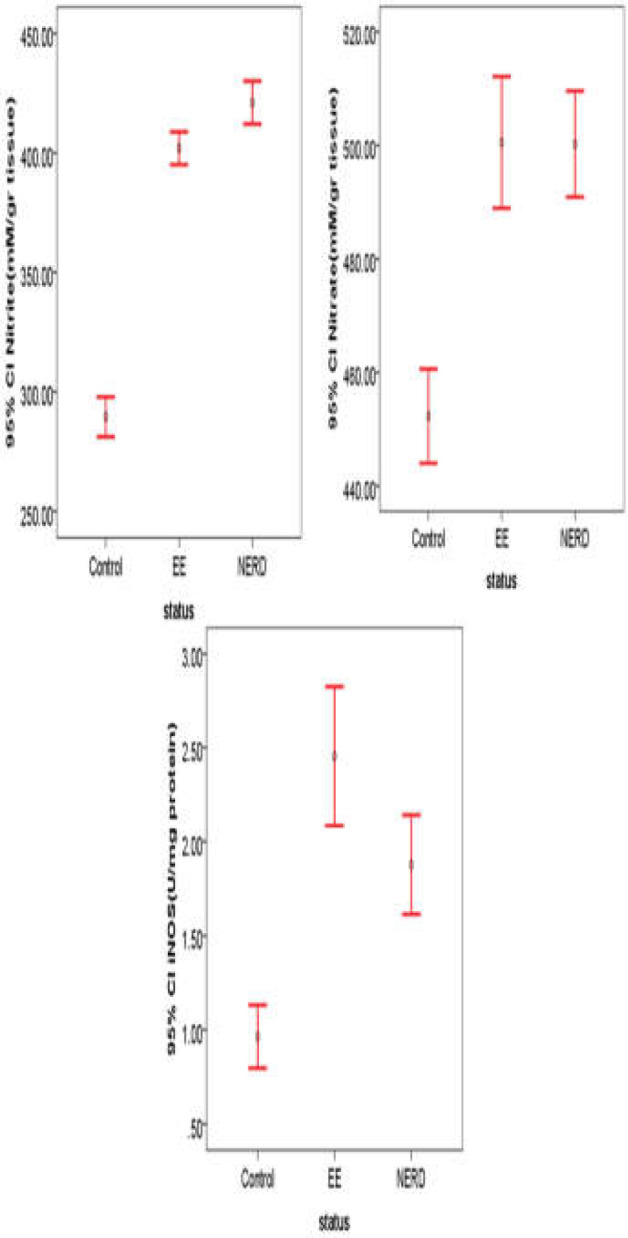
Comparison of biochemical parameters (Nitrite, Nitrate, and iNOS) in control subjects, EE, and NERD. As it is known, the iNOS factor in EE patients was higher than the NERD and control group, while the nitrite and nitrate levels were high in the two groups of patients and had a significant difference with the control group

The mean ages of patients in the EE, NERD, and control groups were 45.62 ± 15.6, 42.48 ± 11.7, and 44.74 ± 13.2 years, respectively. Table 1 demonstrates the demographic information of the patient and control groups, which were relatively similar. Evaluation of biochemical parameters (Table 2) indicated a statistical significance in GERD patients compared with the control group. Nitrite, nitrate, and iNOS factors were statistically different between patients with GERD and control subjects. In multiple comparisons of biochemical characteristics between groups using the Tukey test, as shown in Table 3 and Figure 1, it was found that the levels of iNOS were signiﬁcantly higher (*p*= 0.001) in patients with erosive esophagitis compared with the control group. Moreover, iNOS levels were significantly higher (*p*= 0.01) in patients with EE compared with those with NERD. Tissue levels of nitrite and nitrate were signiﬁcantly higher (*p*= 0.001, *p*= 0.001, respectively) in the patient groups compared with control subjects. This finding indicates that even though their tissue is healthy during endoscopy, NERD patients have high inflammatory factors, similar to patients with EE. In esophageal biopsies from the study patients, the results indicated signiﬁcant increases in nitrite, nitrate, and iNOS. Thus, the development of acute reﬂux esophagitis is associated with increased nitrite, nitrate, and iNOS protein in the esophageal squamous epithelium. The current results indicated that NO and iNOS levels are useful, effective markers in the pathogenesis of GERD. It is also thought that a link between the expressions of iNOS and disease progression exists, though more research is needed to confirm this finding. 

**Table 1:** Demographic characteristics of patients and control subjects

Variable Age (mean ± SD, year)	Erosive esophagitis (n = 27) 45.62 ± 15.6	Non-erosive reflux disease (n = 27) 42.48 ± 11.7	Control subjects (n = 27) 44.74 ± 13.2
Gender (%) Male Female	15 (55.5%) 12 (44.4%)	13 (48.1%) 14 (51.9%)	14 (51.9%) 13 (48.1%)
Marital status (%) Married Single	21 (77.8%) 6 (22.2%)	23 (85.2%) 4 (14.8%)	22 (81.5%) 5 (18.5%)

**Table 2:** Comparison of biochemical characteristics of patients with GERD and control subjects

	N	Mean	Std. Deviation	Std. Error	95% Confidence Interval for Mean Lower Bound Upper Bound	
Nitrite Control (mM/gr tissue) Erosive esophagitis Non erosive reflux disease Total	27 27 27 81	289.48 401.96 421.07 370.84	21.04 17.32 22.73 61.82	4.05 3.33 4.37 6.86	281.16 395.11 412.08 357.17	297.81 408.81 430.07 384.51
Nitrate Control (mM/gr tissue) Erosive esophagitis Non erosive reflux disease Total	27 27 27 81	452.31 500.53 500.24 484.36	20.99 29.30 23.58 33.51	4.04 5.63 4.53 3.72	444.00 488.94 490.91 476.95	460.61 512.12 509.57 491.77
iNOS Control (U/mg protein) Erosive esophagitis Non erosive reflux disease Total	27 27 27 81	0.96 2.45 1.87 1.76	0.42 0.93 0.66 0.93	0.08 0.18 0.12 0.10	0.79 2.08 1.61 1.55	1.13 2.82 2.14 1.97

**Table 3:** Multiple comparisons of biochemical characteristics between groups using Tukey test

Dependent Variable (I) Type (J) Type	Mean Difference (I-J)	P value	95% Confidence Interval Lower Bound Upper Bound	
Nitrite Control Erosive esophagitis (mM/gr tissue) Non-erosive reflux disease Erosive esophagitis Control Non-erosive reflux disease Non-erosive reflux disease Control Erosive esophagitis	-112.47 -131.58 112.47 -19.11 131.58 19.11	0.001 0.001 0.001 0.003 0.001 0.003	-125.80 -144.91 99.15 -32.43 118.26 5.78	-99.15 -118.26 125.80 -5.78 144.91 32.43
Nitrate Control Erosive esophagitis (mM/gr tissue) Non-erosive reflux disease Erosive esophagitis Control Non-erosive reflux disease Non-erosive reflux disease Control Erosive esophagitis	-48.22 -47.93 48.22 0.28 47.93 -0.28	0.001 0.001 0.001 0.999 0.001 0.999	-64.39 -64.10 32.04 -15.88 31.76 -16.46	-32.04 -31.76 64.39 16.46 64.10 15.88
iNOS Control Erosive esophagitis (U/mg protein) Non-erosive reflux disease Erosive esophagitis Control Non-erosive reflux disease Non-erosive reflux disease Control Erosive esophagitis	-1.49 -0.91 1.49 0.57 0.91 -0.57	0.001 0.001 0.001 0.010 0.001 0.010	-1.95 -1.37 1.03 0.11 0.45 -1.03	-1.03 -0.45 1.95 1.03 1.37 -0.11

The mean difference is significant at the 0.05 level

## Discussion

Analyzing cellular events in damage of the esophagus is essential for a further understanding of the disease pathogenesis as well as the development of novel therapies (42). Researchers in the last few decades have shown that NO has a role in tumor process (43, 44). Nonetheless, many studies have shown that NO is a “double-edged sword.” In fact, over-production of NO results in adverse outcomes. NO can cause cytotoxic effects. It has also been said that NO generated by tumor cells may cause the suppression of lymphocytes, consequently preventing the immune system from monitoring malignant cells (45, 46). Therefore, NO was examined in the current study, and the results demonstrated that tissue levels of nitrite and nitrate were signiﬁcantly higher (*p* = 0.001, *p* = 0.001, respectively) in the patient groups compared with the control group. These findings indicate that that even though tissue in patients with NERD is healthy during endoscopy, the inflammatory factors are high in these patients, similar to patients with EE. Thus, nitrite and nitrate have an important relationship with these diseases. Additionally, it was observed that iNOS level was signiﬁcantly enhanced (*p* = 0.001) in the patient groups compared to the controls. Levels of iNOS were also significantly higher (*p*= 0.01) in patients with EE compared with the NERD group. These ﬁndings are consistent with those of McAdam et al. (47), who confirmed enhanced levels of iNOS and NO in adenocarcinoma of the esophagus. McAdam et al. investigated the role of iNOS and NO in DNA damage and NF-kB signaling in cells of the esophagus in a laboratory environment. They showed that refluxed contents including gastric acid and bile salts can motive the iNOS expression and NO production in the esophagus. The iNOS regulated by continuous reflux causes the production of NO and potentially activates NF-kB. The base level of NF-kB is dependent on iNOS, and inhibition of iNOS remarkably decreases NF-kB activity. These results are compatible with the experiment of Ferguson et al. (48), the results of which indicated inflammatory disturbance can cause carcinogenesis with the activation of iNOS. Enhanced expression of this enzyme has been found in adenocarcinoma of the esophagus, BE, and EE. Moreover, Tanaka et al. (49) tested iNOS expression in human squamous cell carcinoma of the esophagus in 57 patients with esophageal cancer. In their study, intracellular NO was considered as a DNA damage agent. The expression of iNOS increased in 50 out of the 57 people. The researchers concluded that an increase in iNOS expression was associated with malignant esophageal cancer. Wilson et al. (50) stated that COX-2 and iNOS are inflammation mediators and regulators of epithelial cell growth. To specify the contribution of iNOS and COX-2 in Barrett's-associated neoplasia, they investigated the expression of these genes in metaplastic Barrett's and esophageal adenocarcinomas. They found elevated iNOS and COX-2 mRNA levels in Barrett's mucosa compared with paired gastric control tissues. These results support the hypothesis that iNOS and COX-2 are involved early and often in Barrett's-associated neoplastic development. In general, among the research reported on GERD illness, the most usual conclusion is enhanced ROS and RNS generation. The accurate role of these reactive species in the pathophysiology of GERD is not yet clear, but if ROS or RNS play a role in tissue injury, then antioxidant remedy is beneficial and can decrease the intensity of illnesses (51).

A number of researchers have tested iNOS expression in human adenocarcinomas of several organs, like the lung, stomach, and prostate [52-54], and in Barrett's esophageal mucosa. Nonetheless, none of these studies have indicated the localization of iNOS in squamous cells or squamous cell carcinomas of the human esophagus. For further future study, histology tests are suggested to determine which cells increase the amount of iNOS and the localization of the increased iNOS expression. Furthermore, the measurement of NF-κB signaling and DNA damage, which are proposed to be downstream of iNOS signaling, could be evaluated to understand the role/function of iNOS upregulation in NERD and EE samples.

The current study investigated the expression levels of iNOS and its downstream molecules in EE, NERD, and control groups. Some in vitro work has been performed in the field to suggest mechanisms by which iNOS pathways contribute to carcinogenic states. While iNOS has been associated with Barrett's metaplasia and esophageal adenocarcinomas for several years, its expression in pathological states before Barrett's are not well investigated in patient samples. 

While the cohort size is limited, the initial results look promising. In the current study, NO and iNOS protein were increased in human esophagitis tissue. The high generation of NO may be associated with immunosuppression in esophageal adenocarcinoma. The adjustment of NO generation by agents may be beneficial in the remedy of esophageal adenocarcinoma. Furthermore, the current results show that NO, iNOS, and nitrosative stress plays a key role in the pathophysiology of GERD. Nonetheless, whether nitrosative stress is the result of inflammation or the cause of it is not yet clear. Therefore, supplementary studies are needed to determine an accurate mechanism.

## Conflict of interests

The authors declare that they have no conflict of interest.
